# Understanding the action of bamocaftor as a potential drug candidate against Cystic Fibrosis Transmembrane Regulator protein: A computational approach

**DOI:** 10.1371/journal.pone.0328051

**Published:** 2025-07-23

**Authors:** Naisarg Patel, Samrat Sarkar, Bala Murali V M, Ishan Kashyap, Premkumar Thiruselvam, Vino Sundararajan, Sajitha Lulu Sudhakaran

**Affiliations:** Integrative Multiomics Lab, School of Bio Sciences and Technology, Vellore Institute of Technology, Vellore, Tamil Nadu, India; Universitat de Vic - Universitat Central de Catalunya, SPAIN

## Abstract

Cystic Fibrosis (CF) is a hereditary condition and can cause permanent respiration problems leading to degraded life quality. The most common variation leading to CF is the F508del variation. CF can cause damage to not just the lungs but also digestive system, pancreas, and other organs. CF decreases the life expectancy of the individuals affected with the constant fear of lung complications. The current methods of treatment include using a combination of drugs to manage the symptoms. The combination of drugs has many side effects and causes damage to other organs like liver, heart or kidneys. In this study, we aim to find a drug that can relieve the symptoms of CF. We began by creating a dataset of potential drug molecules, which was subsequently refined by removing harmful compounds through an ADMET scan. All these compounds were then docked to the mutated Cystic Fibrosis Transmembrane Regulator (CFTR) protein. The compounds with the best docking affinity were Galicaftor and Bamocaftor. A currently approved drug, Ivacaftor was selected as control for the 200 ns Molecular Dynamics (MD) Simulation. The simulation revealed that the CFTR protein remained more stable and compact when complexed with Bamocaftor, when compared to Ivacaftor and Galicaftor. Moreover, the MMPBSA free energy calculations revealed that the free energy of the CFTR-bamocaftor complex is the lowest compared to the other complexes. Our findings reveal the action of bamocaftor on CFTR protein with p.Phe508del variation. However, the absence of in-vivo or in-vitro studies is a limitation, and further experimental validation is necessary to confirm its efficacy and safety.

## 1. Introduction

Cystic Fibrosis (CF) is a fatal hereditary condition brought on by some variations in the Cystic Fibrosis Transmembrane Regulator (CFTR) gene. It is an autosomal recessive genetic disease. This disease has a frequency of 1 in every 3700 newborns in the USA and 1 in every 2500 newborns in the European Countries [1,2]. CF is a genetic disorder that impairs CFTR protein function, disrupting fluid balance and causing thick, sticky mucus to build up in organs. This leads to blockages, infections, and tissue damage. CFTR, part of the ATP-binding cassette transporter family, is expressed in the apical membrane of epithelial cells in organs such as the lungs, intestines, and pancreas, playing a key role in transepithelial fluid homeostasis [3]. The deletion of the phenylalanine (Phe or F) amino acid at position 508 (p.Phe508del) is one of the most common variations occurring in patients suffering from CF [4,5]. Impairing the protein folding mechanism, chloride channel gating, and the stability of CFTR, p.Phe508del becomes a grave variation hence it leads to the misfolding of CFTR giving rise to CF. This defect affects the way salt and fluid are transported through the airways, which can result in long-term infection, inflammation, and even complete loss of lung function [6].

In India, CF is underreported, but an estimated 3,000 children are born with the disease annually [7]. While 40 countries have approved at least one CFTR modulator, most are high-income nations with predominantly White populations. In 2021, 87.6% of 25,497 eligible CF patients in the U.S. received CFTR therapy [8], yet globally, only 12% of an estimated 162,428 people with CF access these drugs [9]. The CF Foundation estimates 40,000 cases in the U.S. and over 100,000 worldwide. Although historically linked to White populations, CF affects people of all racial and ethnic backgrounds. Lifespan for those born with CF between 2018–2022 averages 56 years, with many expected to live into their 60s. Global prevalence is estimated between 144,606 and 186,620.

CFTR modulators are a major advancement in CF treatment, using small molecules to restore defective CFTR function. The key classes, correctors and potentiators, have been especially impactful. Potentiators like ivacaftor improve CFTR activity at the cell membrane, benefiting patients with gating mutations such as G551D. Correctors address folding and trafficking defects, particularly in the p.Phe508del variant. C1 correctors (e.g., tezacaftor, lumacaftor) act on early folding steps, while C2 correctors (e.g., bamocaftor, elexacaftor) complement C1 to enhance efficacy [10,11]. Though amplifiers and stabilizers are still under study, correctors and potentiators form the backbone of CF therapy, offering a targeted approach to the underlying molecular defects [12–14]. These drugs are often used in combination, with the most effective regimens including a C1 corrector, a C2 corrector, and a potentiator, such as in Orkambi, Symkevi, and Trikafta [15,16]. While these therapies offer significant clinical benefits, such as improved lung function and reduced pulmonary exacerbations, they also come with limitations, including high costs, variable patient responses, and potential drug-drug interactions [16].

**Fig 1:**
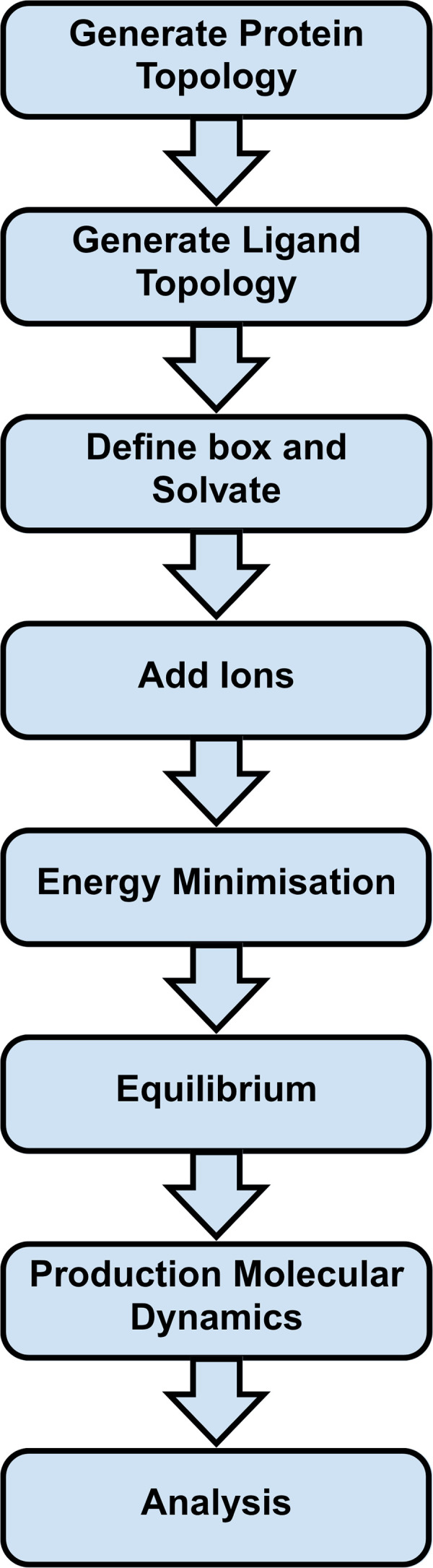
Graphical representation of the research workflow.

This study aimed to identify potential drug compounds targeting cystic fibrosis. A database of possible drug compounds was created and the action of these compounds on the CFTR protein was investigated and reviewed. Compounds were cross-checked with external drug databases and filtered using ADMET profiling via OSIRIS Property Explorer to eliminate toxic, mutagenic, or carcinogenic agents [17,18]. Docking with AutoDock Vina [19,20] predicted binding affinities to the ΔF508 CFTR mutant, and top candidates were selected for 200 ns molecular dynamics simulations. Protein–ligand complexes were assessed through RMSD, RMSF, radius of gyration, hydrogen bonding, and visualized in 3D using VMD [21]. Fig 1 summarizes the workflow.

## 2. Materials and methods

### 2.1. Dataset constructions

Various potential drug molecules belonging to various classes, such as agonists, antagonists, inhibitors, ligands, and others, were collected through open access drug databases. The process was initiated by generating a search query for ‘Cystic Fibrosis’ on Drugbank [22], and more than 200 possible drug molecules were obtained. Among these, compounds that had undergone clinical trials and received approval from public health and safety bodies, such as FDA approvals, were favoured and added to our database. All drugs were cross checked for ongoing clinical trials for CF and cross referenced with additional databases such as PubChem and Kegg drugs. A comprehensive dataset of 200 possible drug molecules was prepared. The 3-D structures of the compounds was downloaded from PubChem and analysed for the presence of double bonds, aromaticity, and additional ring systems. 3-D structure analysis is a key step during drug discovery; it plays a crucial role by exhibiting the role of the drug, its mode of action, and drug-receptor interactions. This chemical formula, PDB structures, and SMILES data were also added to the database. Updated data subsets were formed from the principal dataset by systematically eliminating unfit compounds at each subsequent step of the analysis. This method enhanced the accuracy of decision-making, ensuring that the most reliable and relevant data was used throughout the research.

For scanning the ADMET properties of drugs, the OSIRIS Property Explorer was used. The OSIRIS Property Explorer allowed for the quick computation of several drug-relevant characteristics. Value and color coding provided quick analysis, Red indicated properties that had a high potential for negative consequences, such as mutagenicity, carcinogenicity, or poor intestinal absorption. Green, on the other hand, denoted drug-conformant behaviour. The OSIRIS Software requires the SMILE of the compounds as input, a SMILE or Simplified Molecular Input Line Entry System is the 3-D structure of the compound conveyed in a single line. The results were tabulated and a subset of accepted drug molecules was created.

### 2.2. Protein preparation

The 3-D structure of the mutated CFTR protein (ID: 8EJ1) was downloaded from RCSB in PDB file format [23,24]. The structure was analysed using BIOVIA’s Discovery Studio to correct the residue numbering and fix incomplete residues. The Autodock Tools as a part of MGLTools was used to prepare the protein by adding polar hydrogen and gasteiger charges, this is then saved in the PDBQT format. The predicted binding sites from AutoSite were also analysed and the coordinates for the binding site were generated. This prepared protein file was exported and used henceforth.

### 2.3. Molecular docking

In the current ongoing quest for new therapeutic agents, drug discovery has started utilizing computational methods alongside traditional experimental techniques. The best computational technique that can be used is MDS but due to the time-consuming nature of the simulation, all potential drugs cannot be analysed using MD simulation. The best way to select the highly likely drugs is to first perform molecular docking for all the potential drugs with the drug target, in our case it was the CFTR protein with Δ508F variation. Molecular docking is a type of energy minimisation in which the 3-D structures of the protein and ligand are taken by the algorithm. The ligand is then posed in different conformations in the binding pocket, the bonds formed and the affinity value is calculated. This affinity value and the number and types of bonds formed contribute to the selection of the drug molecules that are the most likely to bind to the mutated protein. The Scripps Research Institute has a very reliable software for molecular docking known as AutoDock Vina. It takes a receptor file, a ligand file and a configuration file containing the box center and size. The receptor here is the mutated protein in a PDBQT file format, the ligands are the potential drug molecules also in a PDBQT file format. The center and size of the box is calculated using the AutoSite program [25] which was also designed by The Scripps Research Institute. The output file contains the best nine conformations of the ligand. The affinity values for all of the nine conformations are also saved in a log file. The best conformation of each potential drug was then analysed in BIOVIA Discovery Studio [26]. The bonds formed between the protein and the drug molecules were analysed in 3-D and 2-D and tabulated.

### 2.4. Molecular dynamics simulations

The standard procedure for MDS using GROMACS [27,28] was implemented as documented by Lemkul et al., 2019 [29]. The first step in MDS was generating a topology file of the protein receptor from the prepared PDB file. For this the *pdb2gmx* function was used with the CHARMM36 force field [30] to generate a topology file, a position restraint file and a post processed structure file. The topology file contains the necessary information of non-bonded and the bonded parameters, which were needed for defining the molecule in simulations. Then a simulation box was created using the *editconf* function with the dimension of 1.0. The next step was to use water as a solvent using the *solvate* function. Then ions were added to the simulation box to neutralize the existing charges in the protein using the *genion* and *grompp* function taking a MDP (Molecular dynamics parameters) file with parameters. Now the solvated box has a net charge of zero and is electroneutral. For the energy minimisation of the system *grompp* and *mdrun* functions were called with another MDP file. The system must be heated to the desired simulation temperature, pressure and the right orientation with respect to the protein must be established, this is done in two steps NVT and NPT using *grompp* and *mdrun* functions with two MDP files. After the equilibration phase, the production MD was run for 200 ns using *grompp* and *mdrun* with an MDP file.

The methodology for protein in water and the protein-ligand complex is very similar, the major difference is preparing the ligand from an SDF file format to the GRO file format, since GROMACS doesn’t support SDF files. The 3-D structure file of the drug molecule was taken from the docked structure of the protein and drug. The hydrogens in the drug molecule structure were explicitly added using the Avogadro program [31] and saved as a MOL2 file. This file was corrected by sorting the bond orders in the file. Then it was uploaded to the CGenFF server for generating the topology in STR format. This file was used to generate the parameter file suitable for Gromacs using cgenff_charmm2gmx.py [32]. Then finally the *editconf* function was used to generate the GRO file. The GRO files for the protein and the drug are combined to get the complex GRO files, which are used for the rest of the simulation.

### 2.5. Principal component analysis

Principal Component Analysis or PCA is a method to visualise the important motions of the protein during a MDS. GROMACS has built in functions to perform the PCA. First the *covar* function was used to calculate a covariance matrix which is mass-weighted, this matrix is used to calculate the eigenvectors. Then the *anaeig* function analyzes the eigenvectors to give an XVG file which can be plotted using gnuplot.

### 2.6. MMPBSA

MMPBSA or Molecular Mechanics Poisson-Boltzmann Surface Area is a method to calculate the free energy of the protein-ligand complex. We used the gmx_MMPBSA software [33]. The MMPBSA was calculated for the last 10 ns of the MD simulations for the complexes. The energies for the protein, ligand and complex were calculated. Using these, the free energy of the binding of protein and ligand was calculated.

### 2.7. Protonation state analysis during MD

To evaluate the stability of protonation states during molecular dynamics (MD) simulations, we performed a residue-level pKa analysis using PROPKA 3.1 [34]. We focused on titratable residues, specifically ARG, ASP, GLU, HIS, and LYS, as they play key roles in modulating electrostatic interactions and contributing to ligand binding affinity. Snapshots of each CFTR–ligand complex were extracted at 0, 50, 100, 150, and 200 ns from the MD trajectories. These frames were converted to PDB format, stripped of water and ions, and analysed individually with PROPKA. The predicted pKa values were recorded for each residue across all timepoints, and mean ± standard deviation was calculated to assess dynamic shifts in protonation propensity.

## 3. Results

### 3.1. Molecular docking

**Fig 2:**
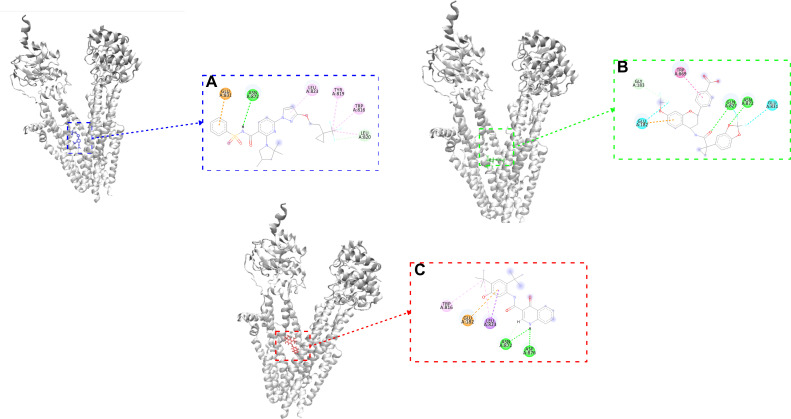
Interaction between the CFTR protein with drug molecules upon Molecular Docking. (A) CFTR-bamocaftor Docking (blue) (B) CFTR-galicaftor Docking (green) (C) CFTR-ivacaftor Docking (red).

The docking results and ADMET properties for all compounds are presented in S1 Table, while the results for the best-performing compounds are detailed in Table 1. Additionally, the 2-D bond diagram is depicted in Fig 2. The best two compounds are Galicaftor with a score of −10.04 kcal/mol and Bamocaftor with a score of −9.097 kcal/mol. The control compound Ivacaftor has a score of −8.247 kcal/mol. In the CFTR-galicaftor complex, GLN-827 and ASN-872 have formed Hydrogen bonds while also forming other bonds like Carbon-Hydrogen, Halogen and Pi-Pi Bond. In the CFTR-bamocaftor complex, ASN-872 has formed Hydrogen Bond while also forming other bonds like Carbon-Hydrogen, Alkyl, Pi-Alkyl and Pi-Anion bonds. In the control CFTR-ivacaftor complex, ASN-872 and ASP-876 have formed Hydrogen Bonds while also forming other bonds like Carbon-Hydrogen, Pi-Alkyl and Pi-Anion bonds. The docking results for all the compounds can be found in S2 Table.

**Table 1:** Results of the molecular docking.

Drug	Docking Score (kcal/mol)	Hydrogen Bonds
Bamocaftor	−9.097	ASN-872
Galicaftor	−10.04	GLN-827, ASN-872
Ivacaftor	−8.247	ASN-872, ASP-876

### 3.2. Molecular dynamics simulations

To confirm the binding activity of the best docked proteins, MD simulations were carried for a period of 200 ns. After the simulation, the trajectory was analysed using various tools included in the GROMACS software package.

**Fig 3:**
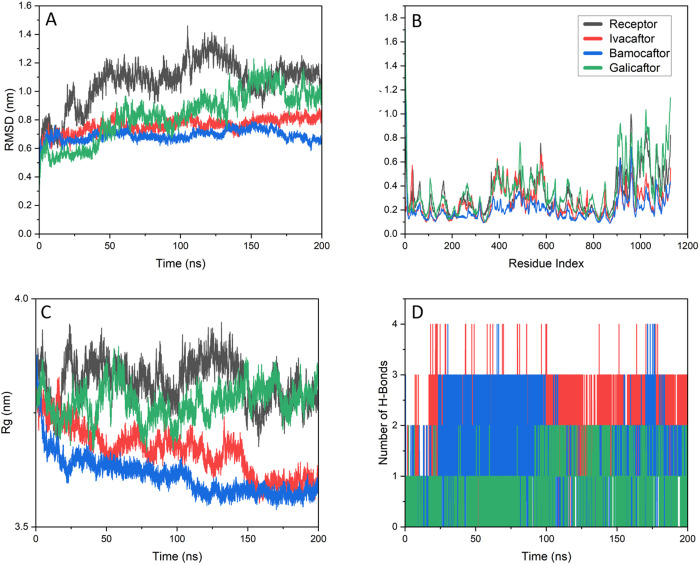
Molecular dynamics simulation results of Bamocaftor (Blue), Ivacaftor (Red) and Galicaftor (Green) with CFTR (Black). (A) Time-dependent RMSD of Backbone, (B) The RMSF of c-α atoms, (C) Radius of gyration vs time, (D) Hydrogen bonds vs Time.

The RMSD, RMSF, Radius of Gyration and hydrogen bonds are shown in Fig 3. The RMSD is a measure of stability since it depicts the deviation of the structure from the starting structure. The RMSD of CFTR-ivacaftor complex and CFTR-bamocaftor complex settled after 100 ns and are stable at 200 ns, averaging to 0.69 nm for Bamocaftor and 0.76 nm for Ivacaftor. The CFTR protein settled after 150 ns and averaged to 1.06 nm while the CFTR-galicaftor complex did not settle even after 200 ns. The RMSF depicts the fluctuations of the residues of the protein. The shape of the CFTR protein is a ‘V’ shape, thus the residues at the ends 350–650 and 900–1126 are showing higher fluctuations. The binding site 650–750 residues is stable in comparison. The Rg or Radius of Gyration depicts the protein compactness throughout the simulation. The CFTR-ivacaftor complex and CFTR-bamocaftor complex have slowly compacted during the 200 ns, averaging to 3.61 nm for Bamocaftor and 3.67 nm for Ivacaftor. The CFTR-galicaftor complex is less compact compared to the control of Ivacaftor with an average Rg of 3.77 nm while the CFTR protein has an average Rg of 3.81 nm. The hydrogen bonds formed between the protein and ligand were also analysed for the duration of the simulation. The CFTR-ivacaftor complex and Bamocaftor-CFTR complexes formed and maintained multiple hydrogen bonds while the CFTR-galicaftor complex failed to maintain hydrogen bonds.

**Fig 4:**
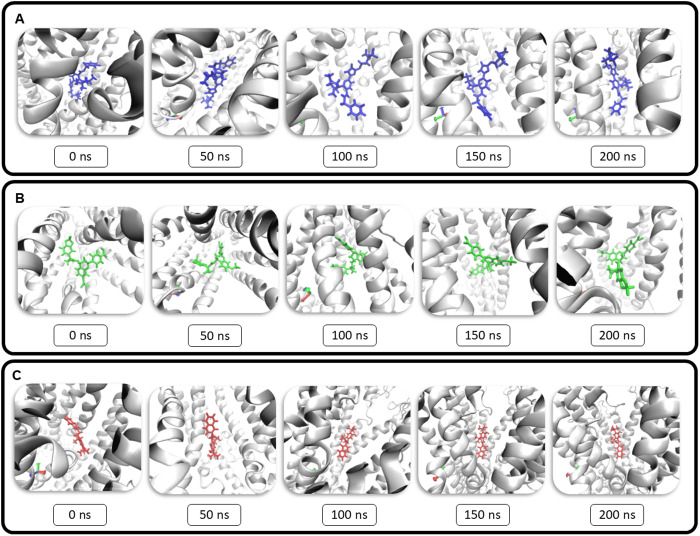
Visualization of the CFTR complexes at 50 ns intervals during Molecular Dynamics simulations. (A) Depict the CFTR-bamocaftor complex, (B) Depict the CFTR-galicaftor complex, (C) Depict the CFTR-ivacaftor Complex.

The trajectory of the simulation was analysed using VMD for every 50 ns and shown in Fig 4, this confirmed that the complex is stable and the ligand does not leave the binding pocket during the simulation.

### 3.3. Principal component analysis

**Fig 5:**
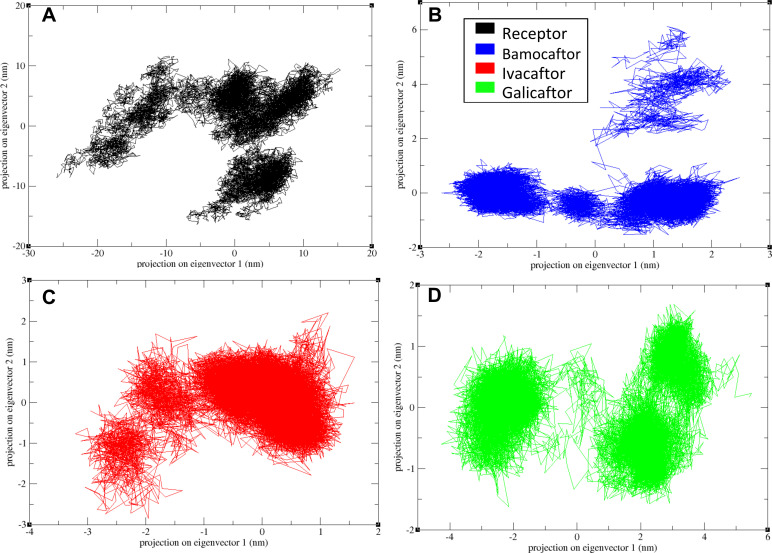
Principal component analysis plot of all three complexes and CFTR protein. Figure A shows the CFTR protein, Figure B shows the CFTR-bamocaftor complex, Figure C shows the CFTR-ivacaftor complex and Figure D shows the CFTR-galicaftor complex.

Principal Component Analysis (PCA) was performed to investigate the dominant motions and overall conformational flexibility of the CFTR protein and its ligand-bound complexes during the simulation. PCA reduces the complexity of the trajectory by identifying major modes of atomic motion (principal components), allowing a comparison of structural fluctuations across systems. As shown in Fig 5, the unbound CFTR protein explores a broad conformational space, indicating high flexibility. In contrast, the CFTR–bamocaftor complex exhibits tightly clustered motion, symmetrically distributed within a confined region, suggesting reduced flexibility and more restricted dynamics. The CFTR–ivacaftor complex shows motion skewed toward the negative side, while the CFTR–galicaftor complex occupies a slightly larger area than bamocaftor but is still more compact than apo-CFTR. The limited and symmetric spread of the CFTR–bamocaftor complex in the PCA plot indicates minimal large-scale fluctuations, which supports the conclusion that this complex is the most stable among those studied.

### 3.4. MMPBSA

The Table 2 shows the results for the MMPBSA done for 190−200 ns of the simulation. The total binding energy of the CFTR-BAM complex is −40.25 kJ/mol, CFTR-GAL complex is −24.71 kJ/mol and CFTR-IVA complex is −26.76 kJ/mol. The Van der Waals Interaction Energy for the CFTR-BAM complex is −52.50 kJ/mol, CFTR-GAL complex −40.57 kJ/mol and CFTR-IVA complex is −38.19 kJ/mol.

**Table 2:** Results of the MMPBSA analysis.

Ligand Name	ΔVDWAALS kJ/mol	ΔEEL kJ/mol	ΔGGAS kJ/mol	ΔGSOLV kJ/mol	ΔTOTAL kJ/mol
BAM	−52.50	−23.10	−75.60	35.36	−40.25
GAL	−40.57	−14.55	−55.12	30.41	−24.71
IVA	−38.19	−14.15	−52.34	25.58	−26.76

### 3.5. Protonation state analysis during MD

We conducted pKa prediction across frames from the MD simulation using PROPKA 3.1 for ARG, ASP, GLU, HIS, and LYS residues. Most residues across all complexes exhibited high protonation-state stability throughout the simulation, with pKa standard deviations below 1.0 [35]. This includes key ligand-proximal residues such as ASP1152 in CFTR–ivacaftor, ARG352 in CFTR–galicaftor, and LYS95 and ARG134 in CFTR–bamocaftor, indicating that their protonation states remained consistent and were not significantly perturbed during the trajectory. Most ARG residues retained high pKa values (around 12.5), indicating that they remained protonated throughout the simulation. ASP and GLU residues consistently exhibited pKa values well below physiological pH, supporting their persistent deprotonated states. A few residues, such as ASP984 and HIS139, showed moderate variance; however, their pKa values remained within a range that does not suggest a change in protonation state under physiological conditions. These results support the validity of the fixed protonation assignments used during system preparation. A summary of per-residue pKa values, including mean and standard deviation over the course of the simulation, is provided in S3 Table.

## 4. Discussions

CF is a prevalent disease in the population worldwide, but it is not easy to diagnose leading to deaths attributed to pneumonia. This is improving with the introduction of fast, cheap and accurate genetic testing methods [36]. The carriers of Cystic Fibrosis can also be identified using the genetic testing methods. The variation p.Phe508del is an in-frame deletion variation leading to the deletion of Phenylaniline [37], this leads to a misfolding of the protein. An individual who is a carrier of p.Phe508del variation has a normal gene and one mutated copy of the Cystic Fibrosis Transmembrane Receptor gene [38]. Though carriers mostly lead a healthy life, they would always be at a risk of respiratory problems or sinus [39]. However, the current methods to treat Cystic Fibrosis are just in the development stage, with the current best treatment being a combination of drugs that increase the number of functional proteins reaching the cell membrane, but also lead to multiple side effects [40,41]. This study aims to propose a drug candidate and understand its mechanism of action on the CFTR protein. To achieve this, a comprehensive database of 200 potential drugs was constructed and analysed. The dataset consisted of the different classes of drugs, potentiators, correctors, amplifiers, and stabilizers. The ADMET scan of these compounds revealed that only 112 compounds were suitable as drugs. The Osiris Property Explorer estimates pharmacokinetic properties using computational models. While these predictions may not fully align with experimentally determined human ADME data, they serve as an excellent tool for filtering out compounds that are incompatible as potential drugs [17]. The remaining 88 drugs in the dataset were rejected due to their potential Mutagenic, Tumorigenic or Irritant activity. The subset of the selected 112 compounds were confirmed to be not Mutagenic, Tumorigenic or Irritant. These 112 compounds were then docked to the mutated CFTR protein using AutoDock Vina. The docking affinities and the bonds formed were tabulated. The more negative the affinity values, the better binding of the ligand with the CFTR protein. The bonds formed were analysed, the most stabilising bond is the Hydrogen Bond formed by an electronegative donor and an acceptor. Ideally a drug molecule should form at least one hydrogen bond with the protein. The other bonds that lead to a more stable compound are pi-pi stacking, pi-alkyl, pi-cation/anion, pi-sigma, and pi-amide interactions [42]. Each contributes to the overall binding energy by attracting electron-rich and electron-poor regions of the ligand and protein, forming a strong complex. The best two candidates after docking were Bamocaftor and Galicaftor.

Although bamocaftor and galicaftor have been evaluated in clinical trials as part of combination therapies for cystic fibrosis, they have not received regulatory approval [43,44]. Following our initial screening of CFTR modulators, we selected these compounds for detailed analysis using molecular docking and MDS to gain insights into their potential mechanisms of action. Blind docking of bamocaftor and galicaftor revealed that both bind in the pocket formed by the CFTR protein. Ivacaftor, a potentiator drug approved for treating cystic fibrosis, also binds in the similar region upon docking. Ivacaftor, with its known mode of action and side effects, has been utilized in the treatment of CF since 2014, making it an ideal control for our study. Other approved modulators like tezacaftor, vanzacaftor and elexacaftor demonstrated a lower affinity when compared to galicaftor and bamocaftor, while for the control group, ivacaftor exhibited a higher binding affinity than deuterated ivacaftor, as presented in S2 Table. Bamocaftor is a small molecule with the formula C_28_H_32_F_3_N_5_O_4_S, it recently completed clinical trials as part of a combination with ivacaftor and tezacaftor. Galicaftor is also a small molecule with the formula C_28_H_21_F_4_NO_7_, it is currently undergoing clinical trials as a Membrane Transport Modulator. Ivacaftor is a small molecule with the formula C_24_H_28_N_2_O_3_, it is approved to be used alone or in combination to treat Cystic Fibrosis. Previous docking studies of ivacaftor with the CFTR protein have shown that it binds to the same site identified in our study, although it interacts with different residues [45]. This discrepancy may result from differences in docking or analysis methodologies. However, in the context of molecular dynamics simulations (MDS), the overall binding site is of greater importance, as specific residue interactions are dynamic and can change over time. While another study investigated the binding of ivacaftor to various blood proteins, highlighting its broader interaction profile, our study focuses specifically on its role as a CFTR modulator [46]. Within this targeted context, our MD simulations revealed that bamocaftor forms the most stable complex with CFTR among the three compounds tested. This is clear from the RMSD, RMSF and Rg values of Bamocaftor which have a lower average than the control Ivacaftor. Bamocaftor also forms hydrogen bonds throughout the simulation to further stabilize the complex. The CFTR-galicaftor complex did not settle during the simulation, this may be due to the inability to maintain hydrogen bonds [47]. From the trajectory analysis it is confirmed that all of the ligands stay in the binding pocket of the protein. The PCA [48] analysis revealed that the CFTR–bamocaftor complex exhibits symmetric and confined motion along the principal components, reflecting stable and coordinated dynamics. In contrast, the CFTR–galicaftor complex displays a semi-symmetric distribution with slightly broader motion, while the CFTR–ivacaftor complex shows an asymmetric spread, primarily shifted toward the positive side of the first eigenvector. Given the inherent symmetry of the CFTR protein, the symmetric clustering observed only in the bamocaftor complex further supports its stabilizing effect on the protein. The MMPBSA or free energy of the binding was calculated by the gmx_MMPBSA software is the energy of the protein and ligand subtracted from the energy of the complex [49]. The negative energies signify that the complex has less energy than the protein and ligand combined, this reveals that the complex is stable. Since the total energy of Bamocaftor (−40.25 kJ/mol) is more negative than Ivacaftor (−26.76 kJ/mol), it shows that the CFTR-bamocaftor binding is very strong and stable.

The analyses of the protonation states of titratable residues as a post-MD simulation step by calculating their pKa values [50]. For each CFTR ligand complex, representative frames from the MD trajectories were extracted and assessed for pKa shifts. Key residues including ASP1152 involved in hydrogen bonding in the CFTR ivacaftor complex, ARG352 in CFTR galicaftor, and LYS95 and ARG134 in CFTR bamocaftor exhibited minimal pKa variation. Their predicted pKa values remained close to the model pH, indicating stable protonation states throughout the simulation. Throughout the analysis, most titratable residues exhibited pKa fluctuations within acceptable limits, with no indication of protonation-state reversal. This finding supports the validity of fixed protonation assignments in our simulation setup. Although constant-pH MD, which enables dynamic protonation state switching, was not employed, the observed pKa tracking across conformational frames provides strong evidence that protonation changes are unlikely to impact binding behavior in this system [51].

Bamocaftor is an investigational CFTR corrector aimed at improving the folding and transport of the CFTR proteins affected by variations such as p.Phe508del. Correctors like bamocaftor enhance the amount of functional CFTR protein at the cell surface, contributing to improved chloride ion transport and alleviation of cystic fibrosis symptoms. Studies have demonstrated its potential efficacy when used in combination with other CFTR modulators like tezacaftor and ivacaftor. Research on similar correctors, as highlighted in trials such as VX-659–tezacaftor–ivacaftor regimens, underscores the promise of combination therapies in significantly improving lung function and quality of life for patients with specific CFTR variations [43]. Vertex conducted a similar study for Elexacaftor or VX-445 which led to slightly better results when compared to bamocaftor [52]. The difference in predicted forced expiratory volume in 1 second (FEV1) was very minor (0.5 points). But Vertex selected elexacaftor over bamocaftor due to a better safety and toxicological profile. Recent studies have revealed the role of elexacaftor as not only a corrector but also as a potentiator. [53,54]. The study also highlights the critical role of ivacaftor in synergizing with elexacaftor. Long term effects of the combination containing elexacaftor are unknown, with studies revealing mental side effects associated with prolonged use of the combination therapy are highly concerning. If such side effects persist, it may necessitate modifications to the treatment regimen. [55]. Understanding the mechanism and action of a drug is essential before its use; however, there is limited knowledge about bamocaftor. Our study employed a systems biology approach to investigate how bamocaftor interacts with the CFTR protein. We uncovered that bamocaftor also binds to the potentiator site, suggesting it might have a dual role similar to elexacaftor, acting as both a corrector and potentiator. Cryo-EM studies have determined that the binding sites for many of the potentiators and correctors are different [12,56]. In our study, both bamocaftor and ivacaftor were observed to bind at a similar location. However, ivacaftor forms strong hydrogen bonds with ASP876 and ASN872, whereas bamocaftor does not interact with ASP876. Further investigation of bamocaftor’s role is warranted through CryoEM studies.

This study primarily relies on molecular dynamics simulations, which, while providing valuable insights into the protein-ligand interactions, do not fully capture important aspects of drug behavior such as bioavailability, off-target effects, or the complexities inherent in living systems. While ADMET predictions offer preliminary information about drug properties, these models may not accurately reflect the true in vivo behavior of the compounds, as they often do not account for factors like metabolism, absorption, or distribution in the body. Furthermore, receptor preparation using MGLTools did not incorporate pH-dependent protonation states, which could influence the electrostatic properties of the binding site and, consequently, affect the accuracy of both docking and MD simulations. Finally, the absence of in vitro or in vivo validation limits the ability to assess the real-world efficacy and safety of the compounds, as laboratory-based or clinical testing is essential for confirming the pharmacological properties and potential toxicities in actual biological systems.

## 5. Conclusion

This study identified potential drugs for Cystic Fibrosis (CF) treatment through in silico analysis. Out of 200 candidates, 112 compounds passed ADMET scans and were docked to the CFTR protein. Bamocaftor and Galicaftor emerged as the top candidates, with Bamocaftor showing the most stable binding and superior binding energy (−40.25 kJ/mol) compared to the control drug Ivacaftor (−26.76 kJ/mol). Bamocaftor’s interaction suggests it might also act as a potentiator, similar to Elexacaftor, although further Cryo-EM studies are needed to confirm this. Comprehensive validation through in-vivo and in-vitro testing is required to validate the results.

## 6. Future prospects

The study can be validated by CryoEM study, inhibitory rate studies, Nuclear magnetic resonance studies of the Bamocaftor complex with the Cystic Fibrosis Transmembrane Regulator protein to confirm the action of the drug.

## Supporting information

S1 Table ADMET Scan Results. (XLSX)

S2 Table Results of Molecular Docking. (XLSX)

S3 Table Results of Post-Simulation protonation analysis. (XLSX)
